# The rise of commodity care

**DOI:** 10.3389/frhs.2025.1611746

**Published:** 2025-07-07

**Authors:** Jacy E. Neczypor

**Affiliations:** ^1^ London School of Hygiene and Tropical Medicine, London, United Kingdom; ^2^ Department of Health Policy, London School of Economics and Political Science, London, United Kingdom; ^3^ Stritch School of Medicine, Loyola University Chicago, Maywood, IL, United States

**Keywords:** commodity care, health systems, direct to consumer (DTC), evidence based medicine, care coordination, informed consent, public- and private sectors

## Abstract

The commoditization of health care under the guise of advanced preventive services and data-driven performance optimization poses risks for patient care and lessons for health systems. This editorial defines and examines “commodity care,” a growing model of direct-to-consumer healthcare characterized by self-referral for advanced diagnostic imaging and/or serologic testing. Promoted as empowering and proactive preventive care, commodity care services frequently operate beyond established clinical guidelines and lack robust evidence to support their clinical utility. Despite appealing marketing claims, these services carry significant risks for patients, including overdiagnosis, false-positive results, and incidental findings that lead to unnecessary interventions that may cause physical, psychological, and financial harms. At the health system level, commodity care contributes to fragmented patient experiences, promotes low-value utilization of healthcare resources, and raises ethical and environmental concerns related to data stewardship and sustainability. Yet, the rising demand for these services also suggests a deeper dissatisfaction among patients with traditional care models, particularly around issues of access, responsiveness, and personalization. Whether driven primarily by shortcomings of conventional healthcare delivery or by shifting patient expectations, the expansion of commodity care warrants careful attention from clinicians, policymakers, and regulators. Defining commodity care is an imperative first step in understanding its implications. This editorial advocates for increased regulatory oversight and rigorous evaluation of emerging healthcare models that increasingly blur distinctions between clinical medicine and consumer-oriented services. Ultimately, the advancement of healthcare technology should support—not erode—the quality, value, and patient-centeredness of care.

## Introduction

From the era of physician house calls to modern concierge medical practices, direct patient contracting models have been a longstanding feature of healthcare delivery. Today, driven by technological advancements and commercialization, consumer-driven health care has rapidly expanded. Companies such as Prenuvo, Function Health, and Neko Health offer services ranging from whole-body MRI (WB-MRI) to extensive serologic testing, marketing them as a means of comprehensive early disease detection and metric-driven health optimization. Through a *scientia potentia est* ethos, these nascent companies tap into a growing demand for more accessible and technology-driven healthcare. Services more closely resemble consumer products than traditional medical care. Beyond technological innovation, commoditization in healthcare reflects increasing dissatisfaction with traditional health systems and patient concerns over the humanity of their care, whereby these so-called disruptor companies position themselves as ideological antipodes to the healthcare status quo: reactionary and disease-centered models of care. With the commercial diffusion of advanced imaging and biomarker optimization, a new model—commodity care—has emerged. The primary objective of this article is to define and critically examine the concept of commodity care. This analysis differentiates commodity care from traditional healthcare models, evaluates its risks and potential merits, and proposes actionable policy recommendations to responsibly guide its growth and integration within contemporary healthcare systems.

The term “commodity care” serves to distinguish these services from primary care, direct primary care, concierge care, and longevity medicine. This neologism describes a self-referral model that shares the purported goals of disease detection and health optimization but fundamentally differs in scope and integration (Table 1). Commodity care lacks key features of traditional primary care models, namely, first-contact access, care coordination, and comprehensive treatment capacity (1). Primary care traditionally serves as a critical platform for population-level health promotion and disease prevention, with evidence demonstrating its cost-effectiveness in reducing overall healthcare expenditures and improving health outcomes (2, 3). In contrast, today's commodity care companies present themselves as cutting-edge preventive care models focused on patient experience and aligned with a culture of data-optimized wellness.

**Table 1:** Comparative characteristics of commodity care, concierge medicine, direct primary care, and traditional primary care models.

Feature	Commodity care	Concierge medicine	Direct primary care	Traditional primary care
Patient-initiated services^a^	Yes	No	No	No
Payment model	Direct payment	Membership	Membership	Insurance/Public funding
Comprehensive services^b^	No	Yes	Yes	Yes
Continuity of care^c^	No	Yes	Yes	Yes
Care coordination^d^	No	Yes	Yes	Yes

^a^ Patients can initiate services independently (e.g., self-schedule imaging or serological testing).

^b^ Comprehensive services include preventive, acute, and chronic care services, typically with diagnostic capabilities (4).

^c^ Continuity reflects sustained patient-provider relationships, coordination, and follow-up over time (4).

^d^ Care coordination is the extent to which a clinician integrates information from multiple sources into a patient's overall care plan (5).

Celebrity endorsements and social media marketing have amplified the visibility of commodity care, increasing demand (6). Stockholm-based Neko Health reportedly conducted over 10,000 scans since its launch and maintains a waiting list exceeding 100,000 individuals (7). Prenuvo, operating in 16 major U.S. cities, has expanded its reach to England, Sweden, and Australia. Commercial enterprises have long permeated the healthcare space, and while profit-driven motives should be scrutinized, they are not outright grounds for objection. The broader impact of commodity care on patients and health systems remains uncertain.

## The doctor’s dilemma: incidentalomas and treating the number

The diagnostics offered by these companies vary and reflect strategies to capture different market segments. Prenuvo and Ezra, for instance, focus on WB-MRI packages, whereas Randox Health markets self-selected bundles of serologic tests, often as tools for self-care. Function Health distinguishes itself by the sheer number of serological tests they offer patients (100+), presented as “data points” for the metric-conscious consumer. Others, like Neko Health, combine both radiologic and serologic testing in its services.

The focus of commodity care on extensive diagnostics poses risks to patients, primarily due to the high prevalence of incidental findings. Evidence suggests that in asymptomatic individuals, up to 32% of WB-MRI scans reveal incidental findings while the tumor detection rate is less than 2% (8, 9). These incidental findings—or incidentalomas—often lead to unnecessary follow-up tests, patient anxiety, and invasive interventions, many of which lack clinical significance but impose substantial costs and potential harm (10). Currently, no major medical society recommends routine WB-MRI for asymptomatic patients or the extensive and prescriptive serological testing promoted by some of these companies. The American College of Preventative Medicine recommends against WB-MRI for oncologic screening in asymptomatic patients (11).

A truism of radiology is that the more you scan, the more you find. However, more findings do not always lead to better outcomes and may reflect lead-time or length-time bias rather than true benefit. South Korea's extensive thyroid screening program in the late 1990s exemplifies this reality. The program led to a dramatic rise in thyroid cancer diagnosis without a reduction in mortality, revealing that many of the detected cancers were clinically insignificant (12). Similarly, indiscriminate serological testing for nonspecific markers such as C-reactive protein offers little clinical utility and demonstrates a negligent use of resources.

Widespread use of other biomarkers is concerning for false positives that can lead to unnecessary care. In November 2024 in the UK, Randox Health promoted a 50% discount on PSA testing, ostensibly to men of all ages. In the context of the widespread media coverage surrounding British Olympic cyclist Chris Hoy's public announcement of stage IV prostate cancer in October 2024, the campaign appears, at best, coincidental (13). From a critical perspective, such practices may be viewed as strategic marketing efforts that exploit the public's availability heuristic following high-profile health disclosures. It is estimated that PSA screening prevents one death from prostate cancer out of 1,000 asymptomatic patients screened and may reduce prostate cancer-specific mortality risk (14, 15). But notably for PSA screening, the risks of false positives, overdiagnosis, and biopsy complications are considerable (16).

An appropriately ordered, normal test result may undoubtedly offer reassurance. However, one wonders what comfort the asymptomatic individual derives from monitoring annual homocysteine levels or how slight changes in leptin every six months offer any meaningful insight into health. Sir William Osler once said, “the good physician treats the disease; the great physician treats the patient who has the disease”. The physician who treats the number can see splendid results in the cholesterol levels of their patient who died weeks ago. A myopic focus on numbers distorts perceptions regarding the broader purpose of healthcare: optimizing patients' functional well-being to help them pursue what they find meaningful in life (17). Chasing biochemical perfection both misguides priorities but also fails to educate patients on the realistic limits of what healthcare can reliably predict or achieve. Replacing clinical nuance and shared decision-making with protocolized biomarker surveillance—often lacking robust evidence—stands in clear opposition to patient-centered care and offers no justification in terms of cost-effectiveness.

Concerns over commodity care extend beyond clinical utility. The environmental impact of advanced imaging is an increasingly important focus. Faster, artificial intelligence-driven MRI protocols may reduce the carbon footprint of individual scans, but these gains could be offset by the resources required to power the artificial intelligence models themselves (18). Additionally, commodity care companies amass vast amounts of personal health information, raising questions about data governance and oversight. Equally troubling is how these services integrate into the broader health system. Prenuvo offers a consultation with a nurse practitioner to review a radiologist-interpreted report, and Function Health provides patients with detailed clinician-generated explanations of their biomarker results; however, findings that require follow-up ultimately task patients with navigating the healthcare system alone. Ideally, they would have access to care or a regular healthcare provider, but this may not always be the case. In fact, a patient may conceivably seek out commodity care precisely because of limited access or responsiveness within the traditional healthcare system. This approach further burdens physicians, who may also be unfamiliar with interpreting uncommon or highly specialized tests, particularly when results are presented outside of a clear clinical context. These concerns reflect a larger, more complex set of issues that extend beyond clinical practice and highlight a broader need for vigilance in addressing the environmental, ethical, and health system impacts of these services.

## Disrupt responsibly

Some aspects of commodity care may offer genuine benefits, particularly in advancing imaging technology. Prenuvo's Project Hercules, launched in 2024, aims to assess the clinical utility of WB-MRIs in detecting significant diseases and identifying novel biomarkers (19). Neko health reports currently running three studies in Sweden (20). While these trials may expand our understanding of advanced imaging and disease pathogenesis, rigorous evaluation and independent validation are crucial, particularly given commercial incentives and potential conflicts of interest. Additionally, innovations in artificial intelligence have shown promise in enhancing imaging-based diagnostic performance. Recent evidence of artificial intelligence-assisted mammography interpretation for example, has demonstrated a reduction in both false-positive and false-negative rates (21). These concomitant advancements in image interpretation and risk stratification may attenuate some of the accuracy-related harms associated with non-targeted screening approaches and improve the overall efficiency of diagnostic pathways. However, caution is warranted, as evidence remains limited, and the theoretical benefits of improved diagnostic accuracy may not outweigh the potential downsides of increased imaging utilization in practice.

The rise of commodity care serves as an opportunity to reflect on systemic flaws in healthcare delivery, including limited access to preventive services, growing patient dissatisfaction with traditional care models, and lack of price transparency—particularly for those in the U.S. These patterns of engagement can be further understood through Andersen's Behavioral Model, which views healthcare utilization as resulting from interactions between population characteristics (predisposing characteristics, enabling resources, and need) and the broader environmental context, including the healthcare system (22). From this perspective, the increasing interest in commodity care reflects not merely consumer preferences but also structural responses to unmet needs within existing systems. Without addressing the underlying system deficiencies that motivate patients to seek alternative models, regulatory efforts aimed solely at commodity care risk focusing on a subset of issues rather than the structural drivers of demand.

The concept of improving clinically significant early detection and empowering patients is not inherently flawed, but whether a tech-first, profit-driven model will engender such change remains questionable. Commodity care, in part, also signals evolving patient expectations for greater control, personalization, and perceptions of preventive care (23). Rather than dismissing these companies outright, governments and healthcare systems must critically assess what aspects, if any, merit adoption. And at the very least, efforts should focus on reimagining healthcare delivery to be more patient-centered.

## Discussion: aligning innovation with evidence

Despite the claims of empowerment and peace of mind, commodity care involves real risks. Over-testing can cause patient harm though false positives, incidental findings of unclear significance, and therapeutic cascades that increase patient anxiety and healthcare costs without improving outcomes. Systematic reviews have reported considerable overuse rates across various diagnostic tests, underscoring the risk commodity care poses in exacerbating inefficiencies and low-value care (24). Commodity care also inherently exacerbates healthcare disparities by creating financial and geographic barriers to access whereby high-cost services in densely populated areas inevitably exclude lower-income and rural populations.

Patient demand also suggests that information asymmetry leaves consumers vulnerable. Marketing approaches that rely heavily on emotional testimonials and selective medical messaging may exploit patient trust and undermine informed consent, ultimately subverting the ideal of perfect agency within the physician–patient principal-agent relationship. Furthermore, information spread through social media platforms can amplify misleading marketing claims, which in turn may downplay the risks of services that lead to overdiagnosis and overuse (25). Stronger regulation of marketing claims and transparent informed consent processes for self-directed care are essential to safeguard patients and ensure they fully understand both the limitations and potential harms of these services. Commercial innovation may play a role in improving health but should not do so at the expense of patient well-being or ethical standards.

To address the direct challenges posed by commodity care, policymakers should implement strategies that promote patient safety, equitable data stewardship, environmental sustainability, and health system integration. First, regulatory frameworks should mandate clear, standardized marketing disclosures—including test accuracy, explicit risks, and diagnostic limitations—in accessible language to enhance transparency. Second, given the widespread commercial use of patients' anonymized health data by direct-to-consumer healthcare companies, policymakers should explore establishing data dividends to ensure patients receive equitable remuneration from their data contributions (26). Third, regulatory frameworks should require comprehensive environmental evaluations of commodity care services, incorporating life-cycle analysis-derived environmental sustainability metrics such as the carbon cost per actionable diagnostic finding (27). Implementing carbon-linked pricing mechanisms could further incentivize ecologically responsible innovations. Finally, to counteract health system fragmentation, policies should require electronic health record interoperability for coordinated follow-up, establish formal referral pathways linking private diagnostic services to public care, and institute cross-subsidization mechanisms, such as surcharges on private diagnostics, to bolster equitable financing for public healthcare systems.

In the meantime, clinicians must be prepared to adapt and respond. This is not the first wave of commodity care—commercial full-body CT centers rose to prominence in the 1990s, only to decline by the mid-2000s—nor will it be the last (28). A *BMJ* umbrella study on incidentalomas quantified the incidence and outcome of secondary findings by imaging modality and organ (29). Data such as these will aid clinicians and patients in weighing the risks and benefits of ordering imaging and support shared decision-making in managing incidentaloma diagnosis. Prioritizing patient education will also be essential in mitigating harm and promoting realistic expectations about what health technology can and cannot offer.

Defining commodity care clearly is essential for effectively assessing its impact. While its appeal may lie in celebrity endorsements, the allure of controlling the unknown, or the promise of health via “perfect” serological markers, the rise of commodity care signals deeper dysfunction. For some, it may reflect dissatisfaction with traditional healthcare over untimely responsiveness, limited access to care, or a rigid system that feels more reactive than proactive. Evidence-based medicine remains fundamentally patient-centered, and innovations emerging from the commercial healthcare space must be held to the same standards. Moreover, health systems must not overlook other critical aspects of quality care, such as responsiveness, equity, and integration (30). We do not have to accept the vision of a Spotify CEO attempting to “[do] for healthcare what Spotify did for music” as the inevitable future of medicine (31). However, we should thoughtfully consider why patients are turning elsewhere and use this insight to guide meaningful improvements within our existing systems. Healthcare delivery need not remain static; meeting the evolving needs of patients requires creativity, flexibility, and responsible innovation grounded in beneficence.

## Data Availability

The original contributions presented in the study are included in the article/Supplementary Material, further inquiries can be directed to the corresponding author.
